# Supplementary material for: The Parenting to Reduce Adolescent Depression and Anxiety Scale: Assessing parental concordance with parenting guidelines for the prevention of adolescent depression and anxiety disorders
Source: PeerJ. 2017 Sep 18;5:e3825. doi: 10.7717/peerj.3825 (PMC5609518; doi:10.7717/peerj.3825)
Supplement: Table S1 [file peerj-05-3825-s001.docx]

# Supplementary Table 1

Table S1

*One-Month Test-Retest Reliability for PRADAS Subscale Scores (N = 175)*

| PRADAS subscale | Pearson’s correlation between baseline and 1-month score |
| --- | --- |
| Parent-child relationship | .65 |
| Involvement | .62 |
| Relationships with others | .32 |
| Family rules | .63 |
| Home environment | .65 |
| Health habits | .72 |
| Dealing with problems | .59 |
| Coping with anxiety | .29 |
| Professional help-seeking | .59 |

*Note.* N = 175. Calculated based on data from baseline to 1-month follow-up in the waitlist control group from sample 1.

*p*s < .001 for all correlations.
