# Supplementary material for: The Parenting to Reduce Adolescent Depression and Anxiety Scale: Assessing parental concordance with parenting guidelines for the prevention of adolescent depression and anxiety disorders
Source: PeerJ. 2017 Sep 18;5:e3825. doi: 10.7717/peerj.3825 (PMC5609518; doi:10.7717/peerj.3825)
Supplement: Table S2 [file peerj-05-3825-s002.docx]

# Supplementary Table 2

Table S2

*Correlations between PRADAS Subscale Scores and Scores on the IPPA and EAC*

| PRADAS subscale | IPPA total score | IPPA trust | IPPA comm. | IPPA alien. | EAC reward | EAC punish | EAC neglect | EAC override | EAC magnify |
| --- | --- | --- | --- | --- | --- | --- | --- | --- | --- |
| Parent-child relationship | .51*** | .51*** | .45*** | .35*** | .44*** | -.42*** | -.46*** | -.20*** | -.21*** |
| Involvement | .43*** | .37*** | .39*** | .36*** | .33*** | -.26*** | -.35*** | -.14*** | -.25*** |
| Relationship with others | .11** | .09* | .12** | .09* | .10** | -.10** | -.09* | -.13*** | -.07 |
| Family rules | .35*** | .31*** | .39*** | .21*** | .41*** | -.18*** | -.36*** | -.09* | -.15*** |
| Home environment | .35*** | .36*** | .32*** | .21*** | .37*** | -.40*** | -.33*** | -.21*** | -.27*** |
| Health habits | .22*** | .16*** | .20*** | .21*** | .18*** | -.22*** | -.20*** | -.25*** | -.22*** |
| Dealing with problems | .51*** | .48*** | .53*** | .32*** | .49*** | -.35*** | -.45*** | -.21*** | -.23*** |
| Coping with anxiety | .40*** | .37*** | .46*** | .22*** | .43*** | -.28*** | -.35*** | -.16*** | -.21*** |
| Professional help-seeking | .28*** | .24*** | .27*** | .22*** | .20*** | -.31*** | -.24*** | -.32*** | -.23*** |

*Note.* IPPA = Inventory of Parent Peer Attachment; EAC = Emotions as a Child Scale; IPPA comm. = IPPA communication subscale; IPPA alien. = IPPA alienation subscale.

* *p* < .05. ** *p* < .01 *** *p* < .001
