# Supplementary material for: The Parenting to Reduce Adolescent Depression and Anxiety Scale: Assessing parental concordance with parenting guidelines for the prevention of adolescent depression and anxiety disorders
Source: PeerJ. 2017 Sep 18;5:e3825. doi: 10.7717/peerj.3825 (PMC5609518; doi:10.7717/peerj.3825)
Supplement: File S1 [file peerj-05-3825-s003.docx]

# Supplementary File 1: Scale Items

Note: in the current study, questions were individually-tailored with the adolescent’s name and gender, as indicated by square brackets below.

This file includes the 79 original items. Items 17 to 22 (*relationships with others* subscale) were removed from the final version of the scale.

Scoring is not provided in the current document due to intellectual property of the scale. Scoring information will be made available on request from the authors.

**FP =** false positive item.

# The Parenting to Reduce Adolescent Depression and Anxiety Scale (PRADAS)

This survey asks you to provide information about your **general** approach to parenting [*insert child participant’s name*]. Please think about your parenting over the *past month* when answering these questions.

**Your relationship with [teenager]**

| **Please indicate how often you do the following.** | Never | Rarely | Sometimes | Often |
| --- | --- | --- | --- | --- |
| 1. I let [my teenager] know that I love [him/her]. |  |  |  |  |
| 2. I make time to ask [my teenager] about [his/her] day and what [he/she] has been doing. |  |  |  |  |
| 3. I can tell when [my teenager] is open to talking with me. |  |  |  |  |
| 4. If I don’t agree with [my teenager]’s opinion, I just tell [him/her] that [he/she] is wrong. **(FP)** |  |  |  |  |
| 5. I make myself available for [my teenager] whenever [he/she] want to talk about [his/her] concerns. |  |  |  |  |
| 6. If [my teenager] wants to discuss a sensitive topic with me, I do it right away regardless of whether other people can hear. **(FP)** |  |  |  |  |
| 7. When [my teenager] looks worried, upset, or angry, I show my concern by asking about [his/her] feelings. |  |  |  |  |
| 8. When [my teenager] is upset, I encourage [him/her] to toughen up. **(FP)** |  |  |  |  |

**Your involvement in [your teenager]’s life**

| **Please indicate how often you do the following.** | Never | Rarely | Sometimes | Often |
| --- | --- | --- | --- | --- |
| 9. We eat dinner together as a family. |  |  |  |  |
| 10. [my teenager] and I do one-on-one activities together that we both enjoy. |  |  |  |  |
| 11. I discourage [my teenager] from participating in any extra-curricular activities (e.g. sports, music), so that [he/she] can focus on [his/her] studies. **(FP)** |  |  |  |  |
| 12. I let [my teenager] decide what [he/she] wants to tell me about school, so I don’t put too much pressure on [him/her]. **(FP)** |  |  |  |  |
| 13. When [my teenager] is going out without me, I know where [he/she] is going, who [he/she] will be with, and what [he/she] will be doing. |  |  |  |  |
| 14. I make it clear to [my teenager] that [he/she] is still dependent on me, as it is important that [he/she] realises this. **(FP)** |  |  |  |  |
| 15. I encourage [my teenager] to try out a variety of activities, to find out what [he/she] is interested in and what [he/she] is good at. |  |  |  |  |
| 16. I increase [my teenager]’s responsibilities and independence over time (e.g. let [him/her] make more decisions about [his/her] life). |  |  |  |  |

**[your teenager]’s relationships with others**

| **Please indicate how often you do the following.** | Never | Rarely | Sometimes | Often |
| --- | --- | --- | --- | --- |
| 17. I encourage [my teenager] to spend time with [his/her] friends. |  |  |  |  |
| 18. I encourage [my teenager] to be kind to others. |  |  |  |  |
| 19. I encourage [my teenager] to participate in a range of social situations to help build [his/her] social skills. |  |  |  |  |
| 20. I encourage [my teenager] to only socialise with people [his/her] own age. **(FP)** |  |  |  |  |
| 21. When [my teenager] has social problems, I talk to [him/her] about the problem and how [he/she] may manage it. |  |  |  |  |
| 22. I encourage [my teenager] to spend time at home with the family on weekends, because [he/she] sees plenty of [his/her] friends during the week. **(FP)** |  |  |  |  |

**Your family rules**

Now we are going to ask you some questions about family rules.

*Note:* By **family rules** we mean any type of established expectations, limits or guidelines for [your teenager]’s behaviour that [your teenager] is aware of.

|  | Yes, for most things | Yes, for some things | No |
| --- | --- | --- | --- |
| 23. Have you set specific, defined rules for [your teenager]’s behaviour? |  |  |  |
| 24. Have you set specific, defined consequences for when [your teenager] doesn’t follow the family rules? |  |  |  |
| 25. Was [your teenager] involved in developing the family rules? |  |  |  |
| 26. Have you talked with [your teenager] about the reasons behind the family rules? |  |  |  |

| 27. Do you ever review or adjust the family rules? |
| --- |
| Yes, all the time. |
| Yes, to adapt to [my teenager]’s maturity and responsibility. |
| No, family rules apply for as long as [my teenager] is under my care. |

| **Please indicate how often you do the following.** | Never | Rarely | Sometimes | Often |
| --- | --- | --- | --- | --- |
| 28. If [my teenager] gets upset with me for enforcing consequences, I let [him/her] get away with breaking the rule to keep peace. **(FP)** |  |  |  |  |
| 29. When I give [my teenager] consequences as a result of breaking rules, I talk to [him/her] about why [his/her] behaviour was not acceptable. |  |  |  |  |
| 30. When [my teenager] breaks the rules, I use consequences to teach [him/her] a lesson, even if it embarrasses [him/her]. **(FP)** |  |  |  |  |
| 31. When [my teenager] behaves well, I reward [him/her] with positive consequences (e.g. praise, attention, or privileges). |  |  |  |  |

**Your home environment**

| **Please indicate how often you do the following.** | Never | Rarely | Sometimes | Often |  |  |
| --- | --- | --- | --- | --- | --- | --- |
| 32. When I have an argument or conflict with [my teenager], I problem solve the issue with [him/her]. |  |  |  |  |  |  |
| 33. When I am angry with [my teenager], I give [him/her] the cold shoulder so that [he/she] learns a lesson. **(FP)** |  |  |  |  |  |  |
| 34. I try to minimise conflict with [my teenager] by ignoring minor issues that irritate me. |  |  |  |  |  |  |
| 35. I tell [my teenager] if I think [he/she] is a lazy, spoilt or selfish person. **(FP)** |  |  |  |  |  |  |
| 36. After I lose my temper with [my teenager], I try to brush it off and forget about it. **(FP)** |  |  |  |  |  |  |
| 37. If I argue with my partner, I make sure that [my teenager] can’t hear. |  |  |  |  | Not applicable,  I don’t have a  partner. | |
| 38. If I argue with my partner, I try to get [my teenager] to be on my side. **(FP)** |  |  |  |  | Not applicable,  I don’t have a  partner. | |
| 39. If I feel angry with others at home, I try to resolve the issue when I’m calm. |  |  |  |  |  |  |

**Health Habits**

| **Please indicate how often you do the following.** | Never | Rarely | Sometimes | Often |
| --- | --- | --- | --- | --- |
| 40. I encourage [my teenager] to eat a healthy, balanced diet, including plenty of fresh vegetables and water. |  |  |  |  |
| 41. Treats (e.g. chips, biscuits, chocolates, or soft drinks) are readily available in our house. **(FP)** |  |  |  |  |
| 42. I have good health habits (i.e. healthy diet, regular exercise, responsible use of alcohol) myself. |  |  |  |  |
| 43. I encourage [my teenager] to go to bed and get up at roughly the same time each day (even on weekends). |  |  |  |  |
| 44. I encourage [my teenager] not to watch TV or use an electronic device in bed before going to sleep. |  |  |  |  |
| 45. I encourage [my teenager] to sleep-in on the weekends if [he/she] hasn’t slept enough during the week. **(FP)** |  |  |  |  |
| 46. I help [my teenager] to engage in physical activity (e.g. transporting [him/her] to sports or dance classes, riding a bike or walking with [him/her], encouraging [him/her] to participate in sports at school). |  |  |  |  |
| 47. I allow [my teenager] to have an alcoholic drink at home to help [him/her] learn to drink responsibly. **(FP)** |  |  |  |  |

| **If you found out that [your teenager] had been misusing alcohol or other drugs, how likely would you be to:** | Very unlikely | Unlikely | Likely | Very likely |
| --- | --- | --- | --- | --- |
| 48. Calmly talk to [him/her] about why [he/she] is using the alcohol or drugs. |  |  |  |  |
| 49. Tell [him/her] how disappointed you are in [him/her]. **(FP)** |  |  |  |  |
| 50. Enforce a punishment [he/she] will not easily forget, so that [he/she] learns not to do this again. **(FP)** |  |  |  |  |
| 51. Seek professional help if you thought it was needed. |  |  |  |  |

**Dealing with problems in [your teenager]’s life**

| **Please indicate how often you do the following.** | Never | Rarely | Sometimes | Often |
| --- | --- | --- | --- | --- |
| 52. I encourage [my teenager] to work towards realistic goals. |  |  |  |  |
| 53. When [my teenager] is stressed or upset, I help [him/her] to learn how to cope with these emotions (e.g. by talking about it with someone [he/she] trusts). |  |  |  |  |
| 54. When talking with [my teenager] about a problem [he/she] has managed, I praise [his/her] problem-solving efforts, rather than focusing on the outcome. |  |  |  |  |
| 55. I give up on tasks that prove to be too difficult. **(FP)** |  |  |  |  |
| 56. I think about the pressures [my teenager] receives from different sources (e.g. school, friends, the media or family members). |  |  |  |  |
| **When [my teenager] faces problems in [his/her] life:** |  |  |  |  |
| 57. I give [him/her] time to talk through the problem. |  |  |  |  |
| 58. I attempt to solve the problem for [him/her]. **(FP)** |  |  |  |  |
| 59. I help [him/her] to break the problem down into smaller, more manageable steps. |  |  |  |  |
| 60. I tell [him/her] it’s not such a big deal, so that [he/she] doesn’t get too upset. **(FP)** |  |  |  |  |
| 61. I encourage [him/her] to think about how [his/her] actions may affect other people. |  |  |  |  |

**Coping with anxiety**

The following section includes questions about anxiety*.*  People may describe anxiety as feeling stressed, nervous, on edge, worried, or scared. Although anxiety may be unpleasant, it can be quite useful in helping us to avoid dangerous situations or solve everyday problems.

| **Please indicate how often you do the following.** | Never | Rarely | Sometimes | Often |
| --- | --- | --- | --- | --- |
| 62. I let [my teenager] avoid situations that make [him/her] anxious, so that [he/she] doesn’t become overwhelmed by anxiety. **(FP)** |  |  |  |  |
| 63. If [my teenager] takes steps to manage [his/her] anxiety, I praise [him/her] for doing it. |  |  |  |  |
| 64. When talking to [my teenager] about things that [he/she] finds anxiety-provoking, I try to remain calm and relaxed. |  |  |  |  |
| 65. I step in to help [my teenager] at the very first sign of stress or anxiety. **(FP)** |  |  |  |  |
| 66. I encourage [my teenager] to face situations that [he/she] finds anxiety-provoking, to help [him/her] learn to manage anxiety. |  |  |  |  |
| 67. I let [my teenager] know the strategies I use when I’m feeling anxious. |  |  |  |  |

| **Have you helped [your teenager] understand that:** | Yes, definitely | Yes, partly | No |
| --- | --- | --- | --- |
| 68. All teenagers experience some level of anxiety. |  |  |  |
| 69. Normal anxiety is useful, as it helps us prepare for situations or perform our best. |  |  |  |
| 70. Anxiety can become a problem if it is severe, long-lasting, or if it interferes with school or other activities. |  |  |  |

**Getting help when needed**

| **If you noticed a persistent change in [your teenager]’s mood or behaviour, how likely would you be to:** | Very unlikely | Unlikely | Likely | Very likely |
| --- | --- | --- | --- | --- |
| 71. Encourage [him/her] to talk to you about what’s going on for [him/her]. |  |  |  |  |
| 72. Encourage [him/her] to ‘snap out of it’ so that it doesn’t become a more serious problem. **(FP)** |  |  |  |  |
| 73. Try to determine whether the change in mood or behaviour is caused by a temporary situation or a more ongoing problem. |  |  |  |  |
| 74. Take [him/her] to a trained mental health professional. |  |  |  |  |
|  |  |  |  |  |
| **Please indicate how likely you would be to do the following.** | Very unlikely | Unlikely | Likely | Very likely |
| 75. I would seek help at the very first sign of any anxiety in [my teenager]. **(FP)** |  |  |  |  |
| 76. I would seek professional help if I was experiencing problems with depression or anxiety myself. |  |  |  |  |

| **If [my teenager] had mental health problems that were interfering with [his/her] life:** | Very unlikely | Unlikely | Likely | Very likely |
| --- | --- | --- | --- | --- |
| 77. I would know where to seek appropriate professional help for [my teenager]. |  |  |  |  |
| 78. [My teenager] would know where and how to seek appropriate professional help for [himself/herself]. |  |  |  |  |
| 79. [My teenager] would be willing to accept professional help. |  |  |  |  |
