# Supplementary material for: The Parenting to Reduce Adolescent Depression and Anxiety Scale: Assessing parental concordance with parenting guidelines for the prevention of adolescent depression and anxiety disorders
Source: PeerJ. 2017 Sep 18;5:e3825. doi: 10.7717/peerj.3825 (PMC5609518; doi:10.7717/peerj.3825)
Supplement: File S2 — Table S3. Logistic regression predicting clinical status (above or below cut-off) on SCAS and SMFQ Scales from PRADAS score. Table S4. Means, standard eeviations, and t-test results comparing PRADAS scores for participants above and below clinical cut-off on the SCAS and SMFQ. [file peerj-05-3825-s004.docx]

**Supplemental File 2: Additional exploratory analyses of participants above and below clinical cut-off scores on the SCAS and SMFQ**

Binary logistic regression analyses were performed to assess the ability of the PRADAS to predict adolescent elevated symptom status (i.e., above or below clinical cut-off scores) on each of the symptom measures. Cut-off scores used to determine these groups were as follows: SCAS-C, ≥ 43 for male adolescents and ≥ 51 for female adolescents; SCAS-P, ≥ 24 for male adolescents and ≥ 26 for female adolescents. These cut-off scores indicate a score greater than or equal to 1.5 standard deviations above the mean, based on published norms from an Australian community sample (available at: <https://www.scaswebsite.com/index.php?p=1_8>). The SMFQ does not have consistent, established cut-off scores for clinical elevation. We used a cut-off score of 8 for the SMFQ-C, which has shown good sensitivity and specificity for detecting diagnosed cases of depression (Angold et al., 1995). For the SMFQ-P, we also used a cut-off score of 8, although it should be noted that there is very limited information published on cut-off scores for the SMFQ-P. We therefore ran additional exploratory analyses with cut-off scores based on the 75^th^, 90^th^, and 95th percentile of our sample. As results did not differ substantially with the varying cut-off scores, and conclusions remained the same, results presented below use a cut-off score of ≥ 8 for both SMFQ-C and SMFQ-P.

Table S3 presents the logistic regression results, which were statistically significant for the SCAS-C, SMFQ-P, and SMFQ-C. For ease of interpretation, we have converted the odds ratios to provide the ratio for a 10-point difference in the PRADAS. This is based on the interquartile range of PRADAS scores (IQR = 10), therefore provides the odds ratio based on scoring at the 25^th^ compared to 75^th^ percentile.

To further explore the relationship between PRADAS scores and symptom elevation status, we conducted *t­-*tests comparing mean PRADAS scores between participants above and below clinical cut-offs on the symptom measures. As shown in Table S4, mean PRADAS scores were significantly lower for participants who scored above the clinical cut-off scores on the SCAS-P (*p* < .001), SMFQ-P (*p* < .001) and SMFQ-C (*p* = .006), with small effect sizes.

*Table S3*

Logistic Regression Predicting Clinical Status (Above or Below Cut-off) on SCAS and SMFQ Scales from PRADAS score

|  | *B* | *SE* | *Wald* | *df* | *p* | Odds Ratio | 95% CI for Odds Ratio | | Cohen’s *d* |
| --- | --- | --- | --- | --- | --- | --- | --- | --- | --- |
|  |  |  |  |  |  |  | Lower | Upper |  |
| **SCAS-P** |  |  |  |  |  |  |  |  |  |
| PRADAS | -0.43 | .12 | 12.68 | 1 | < .001 | 1.54 | 1.21 | 1.94 | 0.24 |
| Constant | 0.74 | .56 | 1.77 | 1 | .18 | 2.11 |  |  |  |
| **SCAS-C** |  |  |  |  |  |  |  |  |  |
| PRADAS | -0.27 | .14 | 3.55 | 1 | .060 | 1.31 | 0.99 | 1.74 | 0.15 |
| Constant | -0.51 | .67 | 0.57 | 1 | .45 | .60 |  |  |  |
| **SMFQ-P** |  |  |  |  |  |  |  |  |  |
| PRADAS | -0.56 | .12 | 21.31 | 1 | < .001 | 1.75 | 1.38 | 2.22 | 0.31 |
| Constant | 1.37 | .56 | 1.22 | 1 | .14 | 3.93 |  |  |  |
| **SMFQ-C** |  |  |  |  |  |  |  |  |  |
| PRADAS | -0.31 | .11 | 7.37 | 1 | .007 | 1.36 | 1.09 | 1.70 | 0.17 |
| Constant | 0.61 | .53 | 1.31 | 1 | .25 | 1.84 |  |  |  |

*Note.* Odds ratios present the relative odds of a subthreshold (non-case) for a 10-point increase in PRADAS scores (the interquartile range of this predictor). The equivalent effect size (Cohen’s *d*) was estimated using the method described in Chinn (2000).

SCAS-P = Spence Children’s Anxiety Scale, parent-report; SCAS-C = Spence Children’s Anxiety Scale, chid-report; SMFQ-P = Short Mood and Feelings Questionnaire, parent-report; SMFQ-C = Short Mood and Feelings Questionnaire, child-report.

*Table S4*

Means, Standard Deviations, and *t*-test Results Comparing PRADAS Scores for Participants Above and Below Clinical Cut-off on the SCAS and SMFQ

|  | *n* | *M* | *SD* | *t* | *df* | *p* | Cohen’s *d* |
| --- | --- | --- | --- | --- | --- | --- | --- |
| **SCAS-P** | 708 |  |  |  |  |  | 0.33 |
| Above cut-off | 158 | 45.20 | 7.44 | 3.63 | 706 | < .001 |  |
| Below cut-off | 550 | 47.62 | 7.37 |  |  |  |  |
| **SCAS-C** | 660 |  |  | 1.89 | 658 | .059 | 0.22 |
| Above cut-off | 96 | 45.80 | 6.35 |  |  |  |  |
| Below cut-off | 564 | 47.36 | 7.63 |  |  |  |  |
| **SMFQ-P** | 708 |  |  | 4.46 | 706 | < .001 | 0.40 |
| Above cut-off | 161 | 44.65 | 8.08 |  |  |  |  |
| Below cut-off | 547 | 47.80 | 7.10 |  |  |  |  |
| **SMFQ-C** | 658 |  |  | 2.75 | 656 | .006 | 0.24 |
| Above cut-off | 200 | 45.95 | 7.43 |  |  |  |  |
| Below cut-off | 458 | 47.68 | 7.45 |  |  |  |  |

*Note.* SCAS-P = Spence Children’s Anxiety Scale, parent-report; SCAS-C = Spence Children’s Anxiety Scale, chid-report; SMFQ-P = Short Mood and Feelings Questionnaire, parent-report; SMFQ-C = Short Mood and Feelings Questionnaire, child-report.

**References**

Angold A, Costello E, Messer S, Pickles A, Winder F, & Silver D. 1995. Development of a short questionnaire for use in epidemiological studies of depression in children and adolescents. *International Journal of Methods in Psychiatric Research,* 5:237-249.

Chinn, S. 2000. A simple method for converting an odds ratio to effect size for use in meta-analysis. *Statistics in Medicine,* 19:3127-3131.
